# Negative regulation of interferon regulatory factor 6 (IRF6) in interferon and NF-κB signalling pathways of common carp (*Cyprinus carpio* L.)

**DOI:** 10.1186/s12917-022-03538-4

**Published:** 2022-12-12

**Authors:** Yaxin Liang, Rongrong Liu, Jiahui Zhang, Yixin Chen, Shijuan Shan, Yaoyao Zhu, Guiwen Yang, Hua Li

**Affiliations:** 1 Shandong Provincial Key Laboratory of Animal Resistance Biology, College of Life Sciences, Shandong Normal University, No. 88 East Wenhua Road, Jinan, 250014 China; 2 College of Fisheries and Life Science, Hainan Tropical Ocean University, No. 1 Yucai Road, Sanya, 572022 China

**Keywords:** Interferon regulatory factor 6 (IRF6), Poly(I:C), *Aeromonas hydrophila*, Interferon, NF-κB, Common carp (*Cyprinus carpio* L.)

## Abstract

**Background:**

Interferon (IFN) regulatory factors (IRFs) is a kind of transcription factors, which play an important role in regulating the expression of type I IFN and related genes. In mammals, IRF6 is not relevant with IFN expression, while zebrafish IRF6 was reported to be a positive regulator of IFN expression and could be phosphorylated by both MyD88 and TBK1. However, the role of IRF6 in the immune response and IFN transcription of common carp is unknown.

**Results:**

In the present study, the cDNA of IRF6 gene (*Cc*IRF6) was cloned from common carp using RACE technique, with a total length of 1905 bp, encoding 471 amino acid residues, which possesses two functional domains of DBD and IAD. Similarity analysis showed that *Cc*IRF6 had more than 50% similarity with IRFs of other vertebrates, and had the highest similarity with grass carp and zebrafish, among which the DBD domain was much more conserved. The phylogenetic analysis showed that *Cc*IRF6 is in the branch of Osteichthyes and has the closest relationship with grass carp. In healthy common carp, the *Cc*IRF6 was expressed in all the examined tissues, with the highest level in the oral epithelium, and the lowest level in the head kidney. After intraperitoneal injection of poly(I:C) or *Aeromonas hydrophila*, the expression of *Cc*IRF6 increased in spleen, head kidney, foregut and hindgut of common carp. Moreover, poly(I:C), LPS, PGN and flagellin induced the expression of *Cc*IRF6 in peripheral leukocytes and head kidney leukocytes of common carp in vitro. In EPC cells, *Cc*IRF6 inhibited the expression of some IFN-related genes and pro-inflammatory cytokines, and dual luciferase reporter assay showed that *Cc*IRF6 reduced the activity of IFN and NF-κB reporter genes.

**Conclusions:**

The present study suggests that *Cc*IRF6 is involved in the antiviral and antibacterial immune response of common carp, and negatively regulate the expression of IFN and NF-κB signalling pathways, which provides a theoretical basis for the study and prevention of fish disease pathogenesis.

## Introduction

Interferon (IFN) regulatory factors (IRFs) is a kind of transcription factors regulating the expression of type I IFN and related genes, and play an important role in immune regulation and inflammatory response [1–3]. Moreover, IRFs also participate in the regulation of cell growth, differentiation and apoptosis, as well as tumorigenesis and metabolism [2, 4–8]. The first member of the IRF family, IRF1, was found in 1988; and till now, there are nine IRF members identified in humans and mice. In addition, IRF10 exists in chickens and fishes, while IRF11 is unique to fishes [9–14]. The N-terminal of IRF is a DNA-binding domain (DBD), which consists of about 120 amino acids. The DBD contains five or six conserved tryptophan repeat regions forming a helix-turn-helix motif, which recognizes and binds the IFN-stimulated response elements (ISRE) to regulate the expression of multiple immune related genes [4, 15, 16]. Compared with the DBD, the C-terminal IRF-related domain (IAD) is more diversiform and critical for the interaction of IRFs with other transcription factors or cofactors, which determines the different transcriptional activities and biological functions of IRFs [2, 17, 18].

Studies in mammals have shown that IRF6 regulates epidermal proliferation and differentiation [19, 20], and mutations in the IRF6 gene can lead to Van der Woude syndrome (VWS) and popliteal pterygium syndrome (PPS) [21], but is not relevant with IFN production. At present, fish IRF6 is rarely studied and has only been reported in zebrafish (*Danio rerio*), half-smooth tongue sole (*Cynoglossus semilaevis*), mandarin fish (*Siniperca chuatsi*), Atlantic cod (*Gadus morhua*) and blunt snout bream (*Megalobrama amblycephala*) [22–27]. The expression level of IRF6 was the highest in the muscle of half-smooth tongue sole, and increased in the spleen, head kidney or liver after stimulation with *Vibrio harveyi*, *Edwardsiella tarda* or megalocytivirus [23]. In mandarin fish, IRF6 had a higher expression level in intestine, and significant increase was observed in skin and intestine at 6 h following the stimulation of poly(I:C), and had an increase at a later stage of infection from 120 hpi in spleen and head-kidney [25]. The IRF6 in blunt snout bream was highly expressed in liver, intestine and gills, and increased in the intestine, liver, spleen, kidney and gills after infection with *A. hydrophila* [27]. However, the transcript of IRF6 in Atlantic cod appeared to have little or no expression in several important immune related tissues such as the spleen and blood, and was non-responsive to LPS as well as poly(I:C) [26]. Furthermore, overexpression of zebrafish IRF6 enhanced the IFN promoter activity and activated the transcription of ISG15, RIG-I and MAVS. In addition, zebrafish IRF6 can be phosphorylated by MyD88 and TBK1, and plays a positive regulatory role in the transcription of IFN [24], which is different from mammalian IRF6.

Common carp (*Cyprinus carpio* L.) is an important freshwater fish cultured in more than one hundred countries, especially in China and many other Asiatic and European countries, which accounts for up to 10% of freshwater aquaculture production worldwide [28, 29]. As common carp is suffering from outbreaks of a wide range of infectious diseases, it is necessary to elucidate the innate immune mechanism of common carp. To date, IRF1, IRF2, IRF3, IRF4, IRF5, IRF7, IRF9 and IRF10 have been reported in common carp, which performed pivotal antiviral and antibacterial immune functions through the regulation of IFN or NF-kappaB signalling pathways [30–36]. In the present study, the full-length IRF6 cDNA sequence (*Cc*IRF6) was cloned from common carp and its amino acid sequence was analyzed. In vivo and in vitro experiments were performed to investigate its antiviral and antibacterial effects. Meanwhile, the regulatory role of *Cc*IRF6 in IFN and NF-κB signaling pathway was clarified by gene overexpression experiment and dual luciflucase reporter gene assay. The present study will reveal the antiviral and antibacterial immune function of IRF6 in common carp, and provide a theoretical basis for the prevention and control of fish infecious diseases.

## Materials and methods

### Experimental challenges of common carp and sampling

The fish used in the present study were obtained from a fish farm (Jinan, Shandong, PR China), which weigh about 200 g per fish and were feeded at 20 °C in a fish feeding system (Aiwen) with purified water in the lab. Six fish were maintained in each 150 L tank and fed daily with commercial fish feed. One week later, four healthy fish were anesthetized and the liver, spleen, head kidney, gills, skin, foregut, hindgut, buccal epithelium and muscle were collected and homogenized using the automatic grinding instrument (Shanghai Jingxin), and total RNAs were extracted using TRIzol reagent (Tiangen). For experimental challenges, forty-eight fish were divided into two groups: twenty-four fish were anesthetized in 100 mg/L MS222 solution (Sigma) and injected intraperitoneally (i.p.) with 500 μl of poly(I:C) solution (2.6 mg/ml, Sigma); twenty-four were also anesthetized but injected i.p. with 500 μl of *A. hydrophila* suspension containing 2.0 × 10^8^ formalin-killed bacteria [34, 37, 38]. At different time points after injection, three fish per group were anesthetized and some immune-related tissues were collected to extract total RNAs. In the challenge experiments, the fish without treatment were as control groups, and denoted by 0 h in the figures. The quality of these RNAs was analyzed by NanoDrop One (Thermo Scientific), and the cDNAs were synthesized using a FastQuant RT Kit (Tiangen). The protocol used in this study referenced our previously published articles [31, 34, 35].

### Cells preparation and culture

The leukocytes of peripheral blood or head kidney (PBLs and HKLs) were isolated from common carp according to the previous studies [34, 39]. In brief, the single-cell suspensions derived from peripheral blood or head kidney were loaded onto the 34 to 51% Percoll density gradients (Sigma) and then centrifugate at 650 g for 30 min. The separated cells were cultivated in the Leibovitz’s L-15 medium at 25 °C. The epithelioma papulosum cyprini (EPC) cells and 293 T cells were cultivated in M199 medium (HyClone) at 25 °C or in DMEM medium (HyClone) at 37 °C. All these medium above were supplemented with 10% foetal bovine serum, 100 U/ml penicillin and 100 μg/ml streptomycin (Gibco).

### Cloning and expression analysis of the *Cc*IRF6

The full-length cDNA sequence of *Cc*IRF6 was obtained using the reverse transcription PCR and 3′- and 5′-Full RACE (rapid amplification of cDNA ends) method (TaKaRa). The IRF6 sequences of different species were aligned and the phylogenetic tree was constructed using the method of ClustalW and Neighbor-Joining respectively in MEGA 6.0 software, and the similarity analysis was performed using the Megalign program in DNAstar 7.0 software. The GenBank accession numbers of these sequences were listed in Table 1. The domains composition of *Cc*IRF6 protein was analysed using the SMART software (http://smart.embl-heidelberg.de). The expression levels of *Cc*IRF6 in different tissues of common carp were detected by real-time PCR in a Rotor-Gene Q PCR instrument (Qiagen) with TransStart Tip Green qPCR SuperMix (TransGen). The relative expression of all genes was calculated using the 2^(−∆∆Ct)^ method, with the ribosomal protein S11 or β-actin as reference gene [31, 34, 35]. The primers used in the present study are listed in the Table 2.

**Table 1:** Protein length and GenBank accession numbers of the IRF6 family members

Species	Protein length	GenBank accession No.
*Ctenopharyngodon idella*	472	AMT92196
*Danio rerio*	492	AAX57954
*Miichthys miiuy*	491	AHB59739
*Corvus cornix*	460	XP_010402606
*Xenopus laevis*	460	NP_001085345
*Mus musculus*	467	AAH08515
*Homo sapiens*	367	AEL89176
*Branchiostoma belcheri tsingtauense*	267	AJA02101

**Table 2:** Primers used in the present study

Primers	Sequences(5′-3′)	Use
IRF6-F	CATCTTTAAGGCCTGGGC	partial sequence amplifying
IRF6-R	GTACAGCTGCTTCAGGTG	
IRF6-5Rout	CTCGCGGCTCTTGTTGAGGGCGCACC	5′ RACE
IRF6-5Rin	CATCCACGCCTTCCTGATACTTCCCCG	
IRF6-3Fout	CTCATCATGGTGCAGGTGGTGCC	3′ RACE
IRF6-3Fin	GCAGCGTGCGGCTGCAGATCTCC	
*Cc*IRF6-Frt	GAAGTATCAGGAAGGCGTGGATG	Real-time PCR
*Cc*IRF6-Rrt	CCGTCGTAGATGAGGTTGAACTC	
*Cc*S11-F	CCGTGGGTGACATCGTTACA	
*Cc*S11-R	TCAGGACATTGAACCTCACTGTCT	
*Cc*IFN-F	TCAATCTCATGGATGCCTCAGAGC	
*Cc*IFN-R	TGGTATTGGGCCACGCATTCTT	
ccTNFα-F	ACAGGTGATGGTGTCGAGGAGGA	
*Cc*TNFα-R	TCTGAGACTTGTTGAGCGTGAAG	
*Cc*ISG15-F	GTGAGCGGTGAAGCCACAGTTG	
*Cc*ISG15-R	GCGAACCGTTATCGGCAGACAG	
*Cc*Viperin-F	GAGAGCCCTTCCTTCACGAGAGAG	
*Cc*Viperin-R	ACTGCCATTGCTAACGATGCTGAC	
*Cc*PKR-F	AGGCTTGATCCACAGAGACCTGAA	
*Cc*PKR-R	CGTTCCAGAAGTTGCACGTCATTG	

### Construction and transfection of recombinant vectors

The coding sequences of *Cc*IRF6 or *Cc*TRIF were amplified by PCR using Phusion High-Fidelity DNA polymerase (PrimeSTAR), and ligated into the pcDNA3.1-EGFP or pEGFP-N1 vectors to obtain the recombinant vectors pcDNA3.1-EGFP-*Cc*IRF6 or pEGFP-N1-*Cc*TRIF, which were extracted from positive clones using an endotoxin-free plasmid isolation kit (Tiangen) and verified by sequencing. The EPC cells were transfected with pcDNA3.1-EGFP-*Cc*IRF6 and/or pEGFP-N1-*Cc*TRIF using the X-tremeGENE HP DNA Transfection Reagent (Roche) [31, 34, 35].

### Dual-luciferase reporter assays

The effects of *Cc*IRF6 on the activation of IFN promoters and NF-κB were performed using Dual-luciferase reporter assays [31, 34, 35]. Briefly, 293 T cells were transfected with reporter gene plasmids pGL-IFN1/2/3-luc or pGL-NF-κB-luc and the recombinant vectors pcDNA3.1-EGFP-*Cc*IRF6 or pEGFP-N1-*Cc*TRIF using Lipofectamine 2000 (Invitrogen). The pGL-Renilla-luc plasmids were transfected together as control. Forty-eight hours later, the Dual-Glo® Luciferase Reagent (Promega) was used to measure the firefly and *Renilla* luciferase activity according to the manufacturer’s instructions.

### Statistical analysis

The differences significance analysis was performed using t-test in GraphPad Prism 6.0, and *P* < 0.05 was considered as significative.

## Results

### Identification of the IRF6 cDNA sequence in common carp (*Cc*IRF6)

**Fig. 1:**
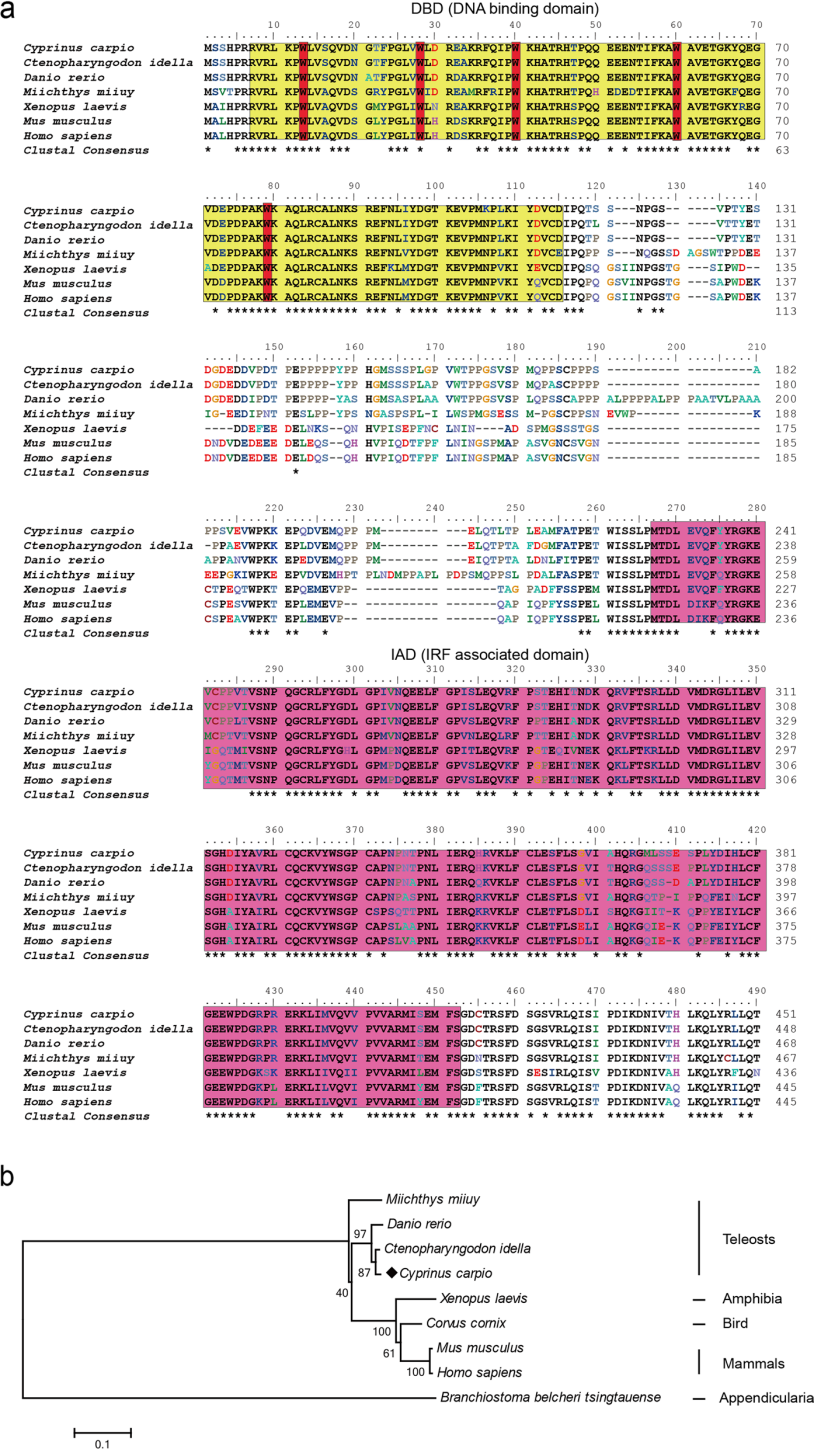
Multiple alignments (**a**) and phylogenetic analysis (**b**) of IRF6 protein sequences in different species. Identical residues are indicated by (*). *Cc*IRF6 is marked with solid diamond (◆). The GenBank accession numbers of the genes are listed in Table 1

The conserved region of IRF6 genes was identified by multi-species sequence alignment, and the primers IRF6-F and IRF6-R designed according to the sequence of *Dr*IRF6 were used to obtain a partial sequence of *Cc*IRF6, with a length of 1138 bp. The 5′ and 3′ RACE were used to amplify the full length of *Cc*IRF6, which was 1905 bp and includes 260 bp 5 ‘UTR, 1416 bp ORF and 229 bp 3’ UTR. The *Cc*IRF6 protein contained 471 amino acids and was composed of a DBD (R7-D115) and an IAD (M228-S413) predicted by SMART software. The DBD domain contained five conserved tryptophan residues, including Trp13, Trp28, Trp40, Trp60 and Trp79 (Fig. 1).

### Amino acid sequence alignment and phylogenetic analysis

Multiple sequence alignment of the IRF6 protein sequences in various species was performed in the present study. The similarity of *Cc*IRF6 with *Ci*IFR6 and *Dr*IRF6 was the highest, up to 94.2 and 90.2% respectively (Table 3). Except for *Bb*IRF6, the DBD domains in all other IRF6 have five tryptophan residues, while the conservation of IAD domains in different species was lower (Fig. 1a). The phylogenetic tree was constructed using the neighbor-joining method, and the results showed that *Cc*IRF6 is in the branch of Osteichthyes, and has the closest relationship with grass carp (Fig. 1b).

**Table 3:** Amino acid identities of *Cc*IRF6 to other IRF6 proteins

Species	Identities (%)
*B. belcheri*	24.7
*C. idella*	94.2
*D. rerio*	90.2
*M. miiuy*	78.4
*X. laevis*	69.4
*C. cornix*	71.4
*M. musculus*	69.4
*H. sapiens*	68.7

### Tissue-specific expression pattern of *Cc*IRF6

**Fig. 2:**
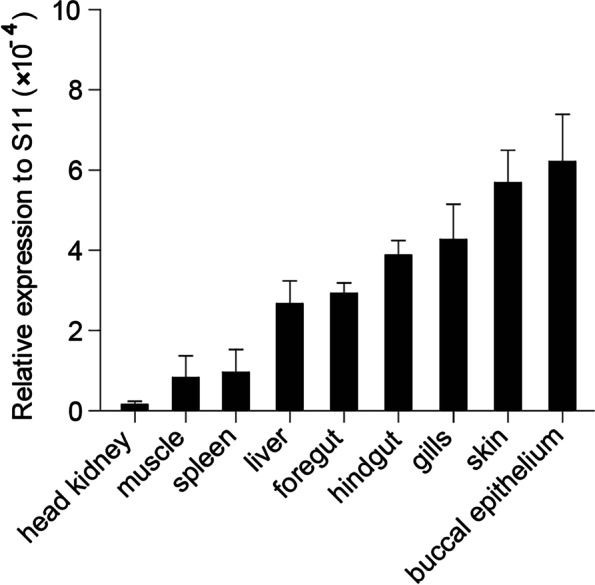
Tissue specific expression of *Cc*IRF6 under normal physiological condition. *Cc*IRF6 expressions in the liver, spleen, head kidney, gills, skin, foregut, hindgut, buccal epithelium, and muscle were determined by Real-time PCR. The expression levels were normalized using the 40S ribosomal protein S11 mRNA. (*n* = 4, mean ± SD)

In order to examined the tissue-specific expression pattern of IRF6 gene in common carp, the expression of *Cc*IRF6 mRNA in nine tissues of healthy common carp was detected by real-time PCR, which is relatively high in oral epithelium, followed by skin, gills, hindgut, foregut and liver, and very low in spleen, muscle and head kidney (Fig. 2).

### The expression of *Cc*IRF6 in response to poly(I:C) and *A. hydrophila* stimulation in vivo

**Fig. 3:**
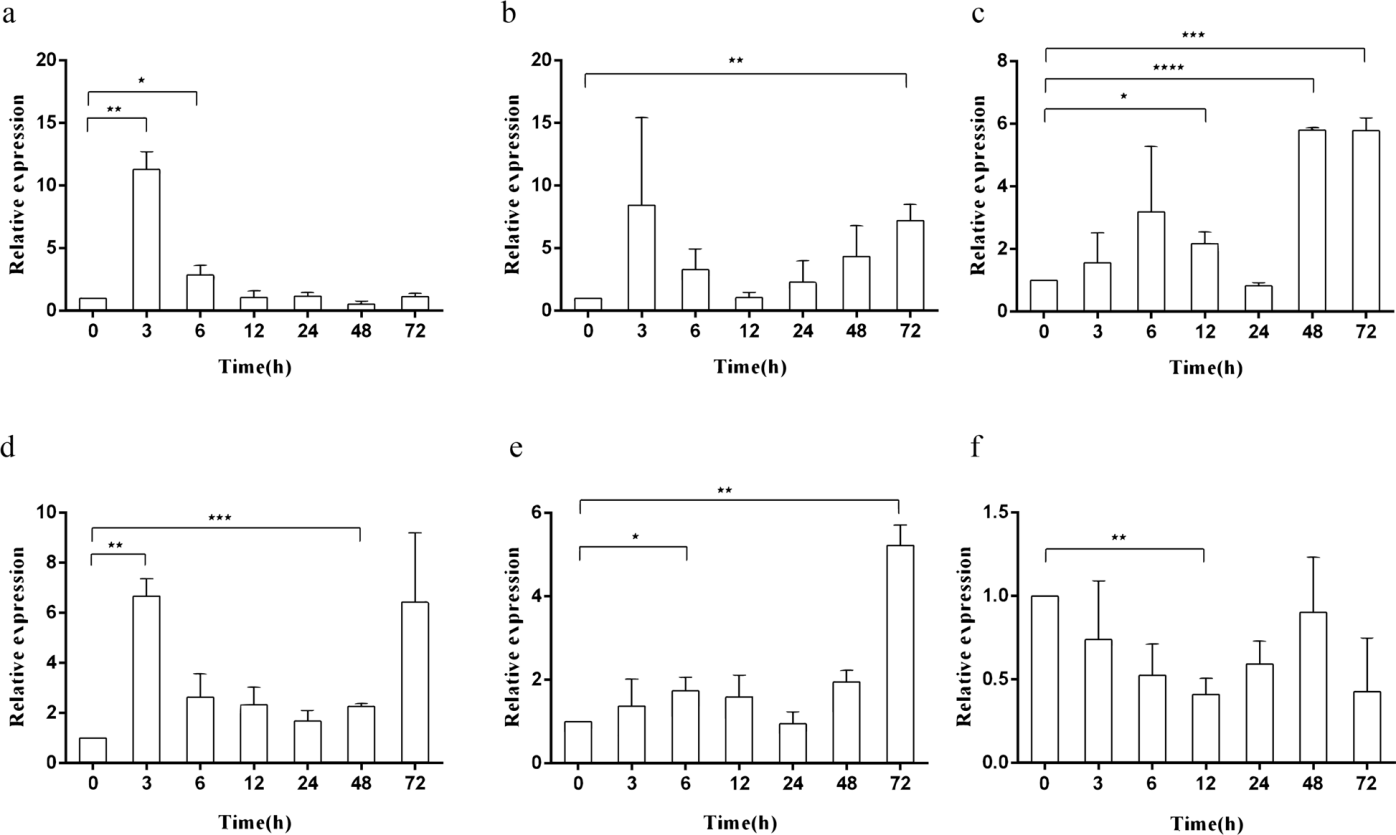
Expression analysis of *Cc*IRF6 in response to poly(I:C) challenge **in vivo.** Total RNA was extracted from liver (**a**), spleen (**b**), head kidney (**c**), skin (**d**), foregut (**e**) and hindgut (**f**) at 0, 3, 6, 12, 24, 48 and 72 h post injection for Real-time PCR. The expression was normalized to the 40S ribosomal protein S11. (*n* = 3, mean ± SD, *P < 0.05)

**Fig. 4:**
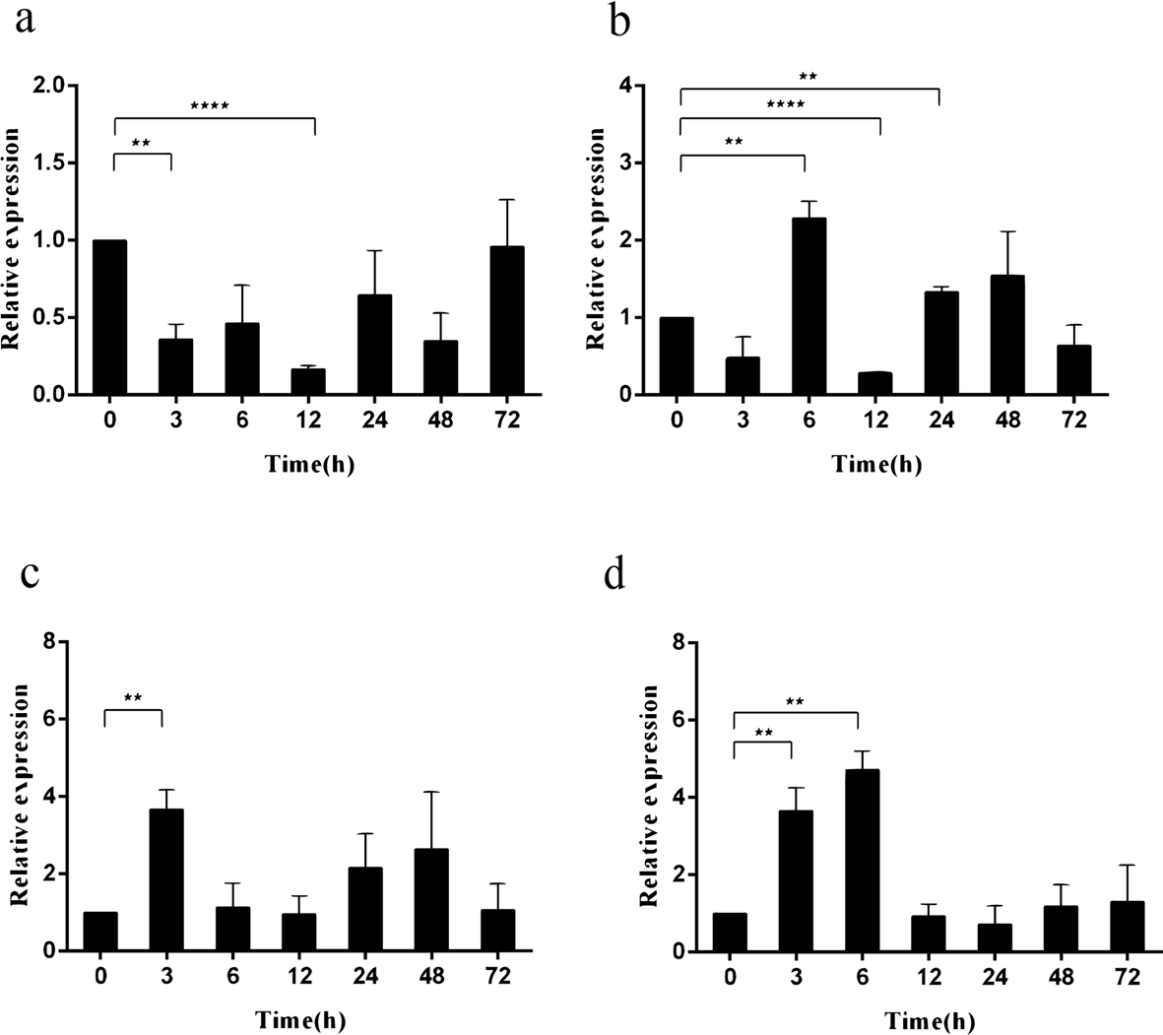
Expression analysis of *Cc*IRF6 in response to *A. hydrophila* challenge in vivo. Total RNA was extracted from spleen (**a**), head kidney (**b**), foregut (**c**) and hindgut (**d**) at 0, 3, 6, 12, 24, 48 and 72 h post injection for Real-time PCR. The expression was normalized to the 40S ribosomal protein S11. (*n* = 3, mean ± SD, *P < 0.05)

Three hours post intraperitoneal injection (hpi) of poly(I:C), the expression levels of *Cc*IRF6 mRNA in liver and skin of common carp were increased by 11.5 and 6.5 times as much as those in the control group, respectively. The *Cc*IRF6 expression increased to 7.2, 5.5 and 9.5 times of the control group in the spleen, head kidney and foregut at 72 hpi, respectively. However, the expression of *Cc*IRF6 in the hindgut decreased to 0.4 times at 12 hpi (Fig. 3). After i.p. injection of *Aeromonas hydrophila*, the expression of *Cc*IRF6 mRNA in the head kidney and hindgut of common carp reached the maximum value at 6 hpi, up to 2.3 times and 4.7 times of that in the control group, respectively, and increased to 3.7 times in the foregut at 3 hpi. However, the expression of *Cc*IRF6 mRNA in spleen decreased by 0.17 times at 12 hpi (Fig. 4).

### Expression of *Cc*IRF6 upon different stimulation in vitro

**Fig. 5:**
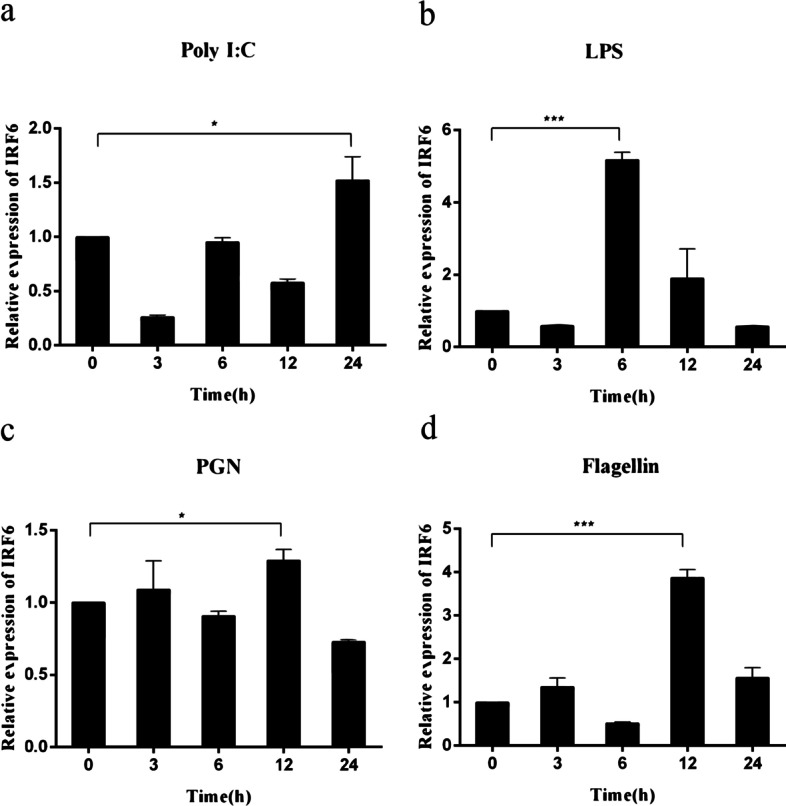
Expression levels of *Cc*IRF6 in the PBLs induced by poly(I:C), LPS, PGN and flagellin. The expression was normalized using the 40S ribosomal protein S11. (*n* = 3, mean ± SD, *P < 0.05)

**Fig. 6:**
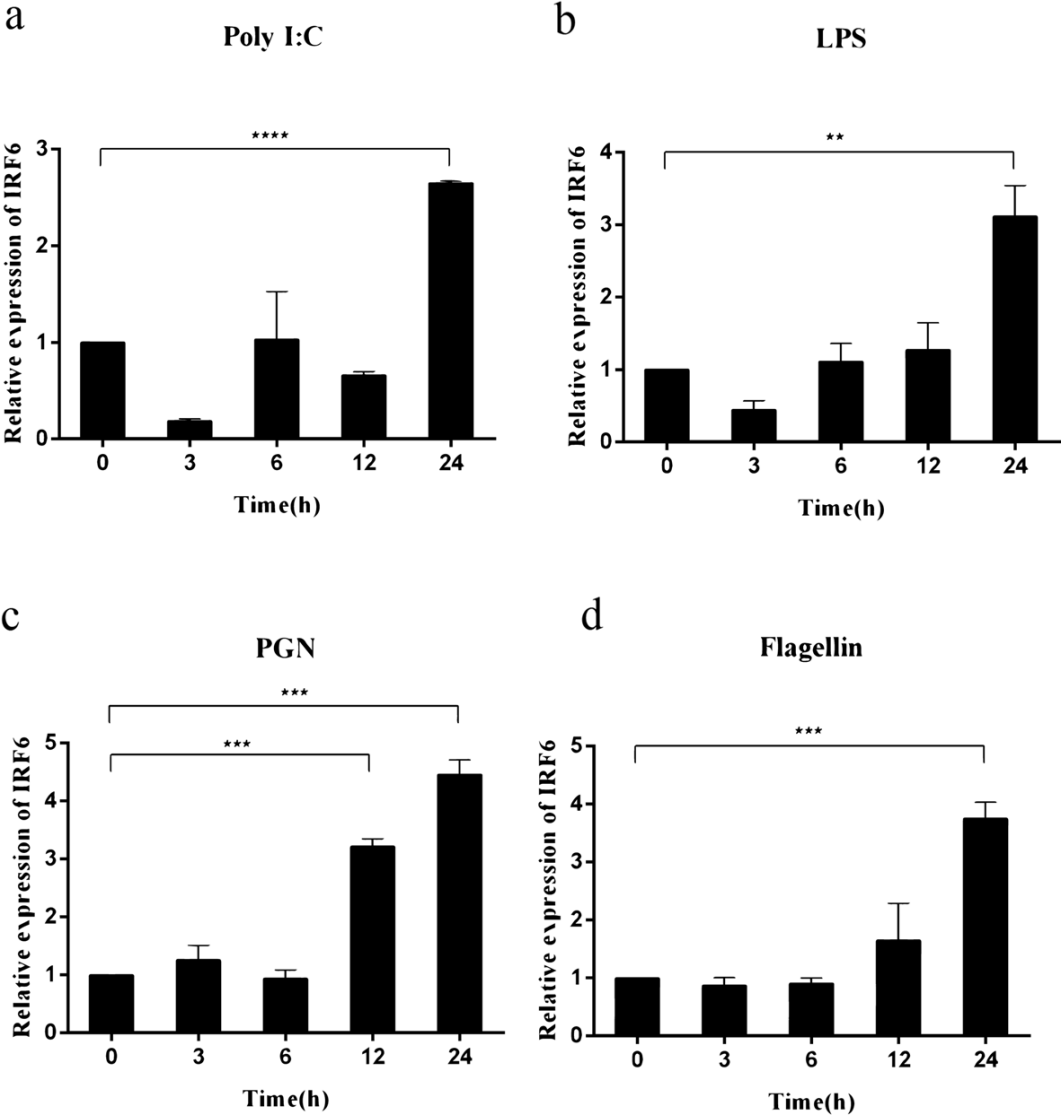
Expression levels of *Cc*IRF6 in the HKLs induced by poly(I:C), LPS, PGN and flagellin. The expression was normalized using the 40S ribosomal protein S11. (*n* = 3, mean ± SD, *P < 0.05)

Twenty-four or 6 hours post poly(I:C) or LPS stimulation, the expression of *Cc*IRF6 mRNA in PBLs of common carp increased 1.5 or 4.6 times as much as that in the control group. 12 h after PGN and flagellin stimulation, the expression levels of *Cc*IRF6 increased to 1.3 and 3.8 times, respectively (Fig. 5). At 24 h after stimulation with poly(I:C), LPS, PGN or flagellin, the *Cc*IRF6 mRNA expression in HKLs of common carp were increased to the maximum with 2.6 times, 3.1 times, 4.4 times and 3.7 times, respectively (Fig. 6).

### Regulation of IFN and NF-κB signalling pathways by *Cc*IRF6

**Fig. 7:**
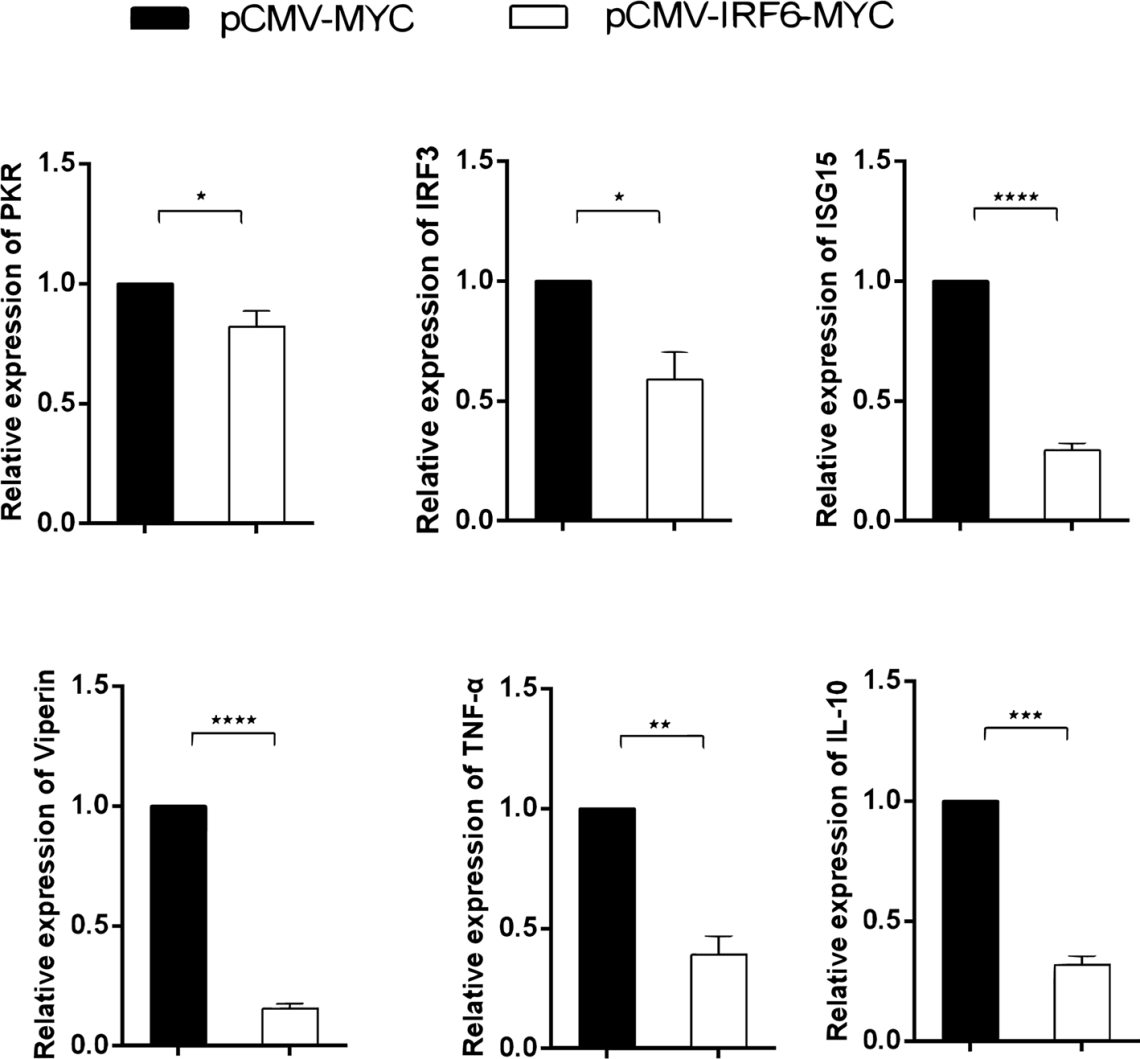
Effect of *Cc*IRF6 on the expression of IFN-stimulated genes and inflammatory cytokines. The expression levels of the PKR (**a**), ISG15 (**b**), Viperin (**c**), IRF3 (**d**), TNF-α (**e**) and IL-10 (f) genes in *Cc*IRF6-overexpressed EPC cells were detected by real-time PCR and normalized to β-actin (*n* = 3, mean ± SD, *P < 0.05)

**Fig. 8:**
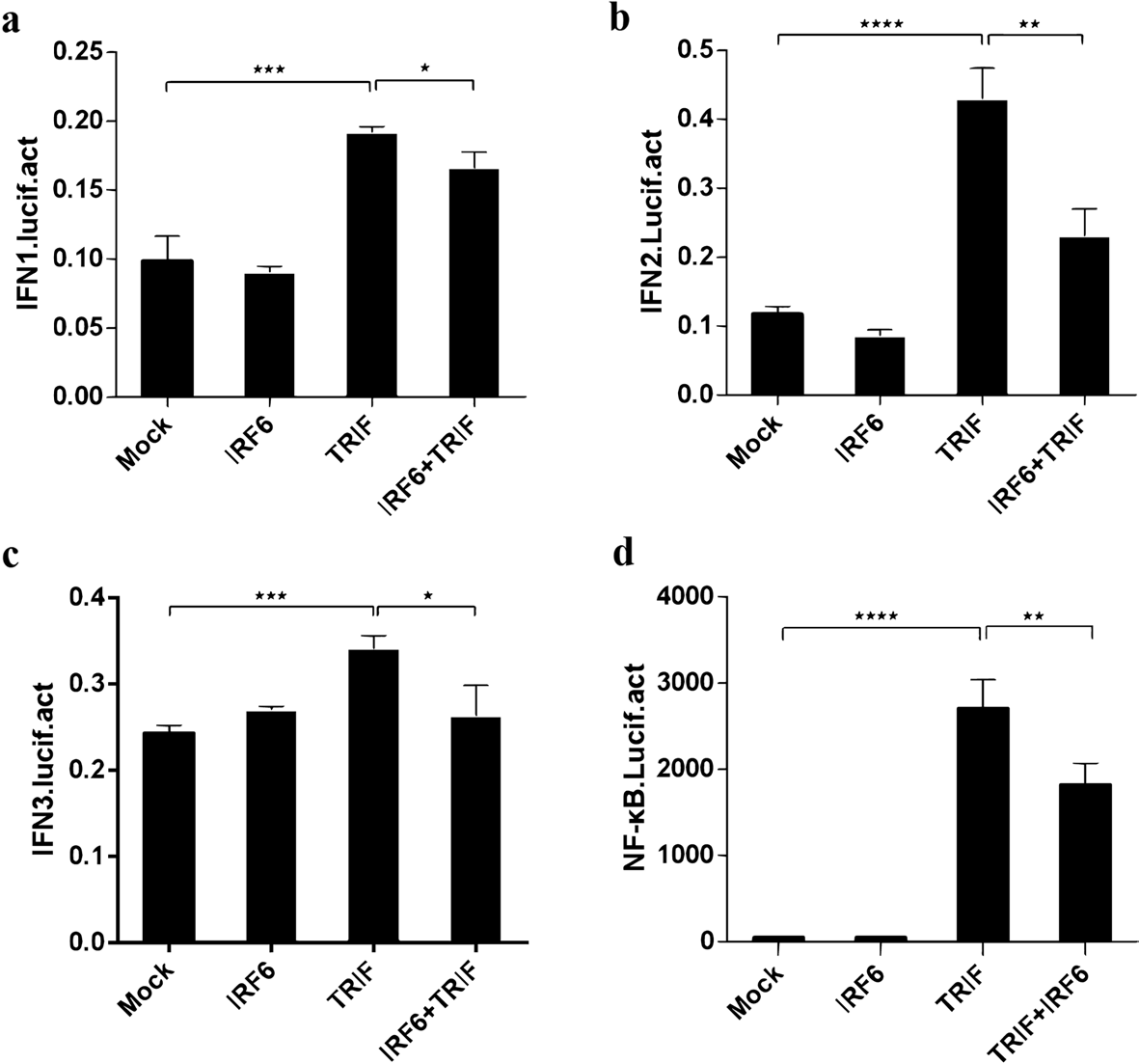
*Cc*IRF6 negatively regulates the IFN and NF-κB pathways. 293 T cells were cotransfected with three IFNs or an NF-κB reporter gene, *Cc*IRF6 and/or TRIF expression plasmids. Luciferase activity was measured after 48 h and determined against Renilla luciferase activity (*n* = 3, mean ± SD, *P < 0.05)

To investigate the role of *Cc*IRF6 in the IFN and NF-κB signalling pathway, the gene expression of PKR, ISG15, Viperin, IRF3, TNF-α and IL-10 was detected after overexpression of *Cc*IRF6 in EPC cells. The results showed that the expression levels of these genes were significantly reduced (Fig. 7). Furthermore, dual-luciferase reporter assays performed in 293 T cells showed that TRIF could activate IFN1, IFN2 and IFN3 promoters, and *Cc*IRF6 reduced the activity of TRIF-induced IFN promoters (Fig. 8A-C). Meanwhile, *Cc*IRF6 also inhibited TRIF-induced NF-κB activity (Fig. 8D).

## Discussion

Most of the organisms control the pathogens invasion by a series of inflammatory factors in the early stage of infection. IRFs were first discovered as transcription factors that regulate IFN expression, and in recent years, studies have shown that IRFs has multiple functions. In addition to the important role in innate and adaptive immunity, IRFs also participates in regulating the development and maturation of the immune system, as well as cell growth [2, 4, 40, 41]. With the rapid development of fishery economy, there are more and more studies on IRFs in teleost fish.

Both mammalian and fish IRFs have DBD domains, which are responsible for binding ISRE/IRF-E sequences on target genes [42]. In this study, the N-terminal region of *Cc*IRF6 has a DBD domain, which is highly similar to the DBD of other vertebrate IRFs, suggesting that the function of IRFs may be relatively conserved in evolution. The DBD domain of IRF2 has six tryptophan residues, while IRF4, IRF9, IRF10 and IRF6 all have five tryptophan residues [31, 34–36]. Although IRF10 and IRF6 are not the same family, their tryptophan residues are all located in Trp13, Trp28, Trp40, Trp60 and Trp79. The C-terminal of IRFs have two types [6]: IAD1 was originally found in IRF8 and is present in all members of the IRF family except IRF1 and IRF2; IAD2 exists only in IRF1 and IRF 2[6]. In this study, *Cc*IRF6 have an IAD1 domain.

In this study, the IRF6 amino acid sequences similarity between common carp and other species up to more than 70%, which indicates the evolutionary conservatism of vertebrate IRFs and reflects the essential role of IRF family members in organisms. Phylogenetic analysis showed that the 11 members of IRF family were divided into four subfamilies, namely IRF1 subfamily, including IRF1, IRF2 and IRF11; IRF3 subfamily, including IRF3 and IRF7; IRF4 subfamily, including IRF4, IRF8, IRF9 and IRF10; IRF5 subfamily, including IRF5 and IRF6. *Cc*IRF6 belongs to the IRF5 subfamily, on the same branch as grass carp and zebrafish.

Mammalian IRF6 is both a transcriptional regulator in keratinocyte differentiation and can also control the transformation from proliferation to differentiation by activating differentiation-related genes [43, 44]. IRF6 is expressed in a variety of human organs and plays an important role in the expression of TLR2-mediated inflammatory cytokines in human oral epithelial cells [45]. The present study found that the expression of *Cc*IRF6 is highest in the oral epithelium of healthy common carp, which is the first line of defense against pathogen invasion. Therefore, IRF6 may play a key role in the immune system of mammals and common carp. In addition, the expression level of IRF6 in muscles of half-smooth tongue sole is the highest, implied that IRF6 may be involved in the regulatory process of tissue connecter formation of fish [23].

Poly(I:C) is an inducer of fish IFN, ISGs and a variety of IRFs, and can also induce the expression of TLR3 and TLR22 [46–48]. *A. hydrophila* is a pathogen of many aquatic organisms, which is widely distributed in natural water and can induce the expression of PRR, IFN and IRFs. In this study, the temporal changes of *Cc*IRF6 mRNA expression in immune-related tissues of commom carp were investigated after stimulation with poly(I:C) and *A. hydrophila*. After poly(I:C) stimulation, the expression of *Cc*IRF6 is increased in various immune-related tissues, including liver, spleen, head kidney, skin and foregut. After *A. hydrophila* infection, the expression of *Cc*IRF6 increased in the head kidney, foregut and hindgut. Similarly, in liver, spleen and head kidney of half-smooth tongue sole, the expression of IRF6 increased after stimulation with *E. tarda* and megalocytivirus [23], indicating that IRF6 is involved in the process of antiviral and anti-bacterial immune responses in fish.

The immune system of bony fish is in a special position in evolution, with the differentiation of natural and adaptive immunity. T and B lymphocytes appear in peripheral blood of bony fish, which play an important role in the immune process [49–52]. Meanwhile, the head kidney is an important immune organ of fish, rich in a variety of immune cells. Therefore, in this study, the peripheral blood leukocytes (PBLs) and head kidney leukocytes (HKLs) were isolated from common carp and stimulated by different PAMPs, such as poly(I:C), LPS, PGN and flagellin. The results showed that the expression of *Cc*IRF6 were significantly increased, indicating that *Cc*IRF6 was involved in the immune response process of antiviral and anti-bacterial in common carp.

As transcription factors, various IRFs play an important role in the regulation of IFN and ISGs expression. However, mammalian IRF6 has nothing to do with the production of IFN, but is involved in the formation of tissue ligand and controls the development of lingual dorsal filamental papillae [53], and its gene mutation can lead to congenital chromosomal dominant genetic disease VWS or PPS [21]. Studies in zebrafish suggest that IRF6 plays a positive regulatory role in the expression of IFN. Overexpression of IRF6 in zebrafish can enhance IFN promoter activity and activate transcription of ISG15, RIG-I and MAVS in host cells [22]. Differently from IRF6 of zebrafish, the expression levels of PKR, ISG15, Viperin, IRF3, TNF-α and IL-10 were significantly reduced after overexpression of *Cc*IRF6, and *Cc*IRF6 reduced the activity of TRIF-induced IFN promoters and NF-κB. Therefore, the function of IRF6 is not conserved in the evolution of lower vertebrates to mammals. Although mammalian IRF6 is unrelated to the production of IFN, fish IRF6 participates in the innate immune process and plays a regulatory role in the transcription and phosphorylation of IFN.

## Conclusions

In the present study, the full-length IRF6 cDNA sequence (*Cc*IRF6) was cloned from common carp and its amino acid sequence was analyzed. We found that various bacterial and viral PAMPs induced the expression of *Cc*IRF6 in vivo and in vitro. Furthermore, *Cc*IRF6 inhibited the expression of some IFN-stimulated genes and inflammatory cytokines, and reduced the activity of IFN promoter and NF-κB. Taken together, these results suggest that *Cc*IRF6 is involved in the antiviral and antibacterial immune response of common carp, and negatively regulate the IFN and NF-κB signalling pathways. On the one hand, this study is helpful to understand the evolution of immune system from lower fish to higher mammals; on the other hand, it provides a theoretical basis in the prevention and control of fish infecious diseases, for example, through providing drug targets or new vaccine research strategies.

## Data Availability

The accession number of *Cc*IRF6 in this article is ON256314, which is available in the GenBank (https://www.ncbi.nlm.nih.gov/nuccore/ON256314).

## Abbreviations

IRF: Interferon regulatory factor
DBD: DNA-binding domain
IAD: IRF associated domain
ISRE: IFN-stimulated response element
VWS: Van der Woude syndrome
PPS: Popliteal pterygium syndrome
ISG15: IFN-stimulated gene 15
RIG-I: Retinoic acid-inducible gene I
MAVS: Mitochondrial antiviral signaling protein
MyD88: Myeloid differentiation primary response gene 88
TBK1: TANK binding kinase 1
i.p.: Intraperitoneally
PBL: Peripheral blood leucocyte
HKL: Head kidney leukocyte
EPC: Epithelioma papulosum cyprinid
RACE: Rapid amplification of the cDNA ends
SMART: Simple modular architecture research tool
PAMP: Pathogen associated molecular pattern
NF-κB: Nuclear factor kappa B
